# Induction of Heme Oxygenase-1 by 15d-Prostaglandin J_2_ Mediated via a ROS-Dependent Sp1 and AP-1 Cascade Suppresses Lipopolysaccharide-Triggered Interleukin-6 Expression in Mouse Brain Microvascular Endothelial Cells

**DOI:** 10.3390/antiox11040719

**Published:** 2022-04-06

**Authors:** Chien-Chung Yang, Li-Der Hsiao, Ya-Fang Shih, Ching-I Chang, Chuen-Mao Yang

**Affiliations:** 1 Department of Traditional Chinese Medicine, Chang Gung Memorial Hospital at Tao-Yuan, Kwei-San, Tao-Yuan 33302, Taiwan; r55161@cgmh.org.tw; 2 School of Traditional Chinese Medicine, College of Medicine, Chang Gung University, Kwei-San, Tao-Yuan 33302, Taiwan; 3 Department of Pharmacology, College of Medicine, China Medical University, No.91, Hsueh-Shih Road, Taichung 40402, Taiwan; lidesiao@livemail.tw (L.-D.H.); shihyafang@mail.cmu.edu.tw (Y.-F.S.); zyy0427@tmu.edu.tw (C.-I.C.); 4 Department of Post-Baccalaureate Veterinary Medicine, College of Medical and Health Science, Asia University, Wufeng, Taichung 41354, Taiwan

**Keywords:** HO-1, 15d-PGJ_2_, bEnd.3, ROS, interleukin-6, lipopolysaccharide, Sp1, AP-1

## Abstract

Heme oxygenase-1 (HO-1) has been shown to exert antioxidant, anti-inflammatory, and anti-apoptotic effects in various types of cells. Therefore, the induction of HO-1 is an excellent rationale for the development of protective drugs. 15-Deoxy-Δ^12,14^-prostaglandin J_2_ (15d-PGJ_2_) can modulate the expression of antioxidant defense proteins and be beneficial for neuroinflammation. Brain endothelial cells play an important role in the pathophysiology of brain disorders. Whether 15d-PGJ_2_ can induce HO-1 expression and protect against the inflammatory responses in mouse brain microvascular endothelial (bEnd.3) cells remains unclear. Here, we reveal that 15d-PGJ_2_ stimulated HO-1 protein and mRNA expression in a time- and concentration-dependent manner in bEnd.3 cells, which was attenuated by diphenyleneiodonium chloride (DPI) and MitoTempo. Thus, activation of NADPH oxidase (NOX)- and mitochondria-derived reactive oxygen species (ROS) mediated 15d-PGJ_2_-induced HO-1 expression. ROS generation could cause phosphorylation of protein kinase C (PKC)δ, leading to HO-1 expression, which was suppressed by Rottlerin (selective inhibitor PKCδ), DPI, and MitoTempo. We further demonstrated that phosphorylation of c-Jun N-terminal kinase (JNK)1/2 participated in 15d-PGJ_2_-upregulated HO-1 expression, which was blocked by SP600125 or Rottlerin. Moreover, 15d-PGJ_2_-induced HO-1 expression was mediated through the activation of c-Jun (a subunit of activator protein 1 (AP-1)) and specificity protein 1 (Sp1), leading to their interaction with the HO-1 promoter, revealed by chromatin immunoprecipitation assay, which was attenuated by SP600125, Mithramycin A, or Tanshinone II A. We further verified the anti-inflammatory effect of HO-1 expression. Our results showed that 15d-PGJ_2_-induced HO-1 could mitigate the lipopolysaccharide-triggered interleukin-6 expression and secretion, as measured by an ELISA assay kit. These results suggest that 15d-PGJ_2_-induced HO-1 expression is mediated through the activation of NOX- and mitochondria-derived ROS-dependent PKCδ/JNK1/2/Sp1 and the AP-1 signaling pathway and protects against inflammatory responses in bEnd.3 cells.

## 1. Introduction

Microvascular endothelium, one of the main elements of the blood–brain barrier (BBB), has been proposed to maintain the integrity of BBB and homeostasis, provide support and protection for the neurons, and participate in the immune and repairment responses to brain injury. One common feature of brain injuries is the presence of brain edema resulting from BBB breakdown. Many reports have implicated that recruitment of inflammatory cells, apparently via breakdown of BBB, participates in a variety of brain pathogenesis. Thus, the failure of the tight junction of endothelium is critically involved in the pathogenesis and degeneration of the brain [1,2,3,4]. Several pieces of evidence have shown that endothelium may contribute to the brain inflammatory process and produce inflammatory mediators, including neurotoxic substances and cytokines. Endothelial cells are thought to be responsible for neural death and brain injuries after exposure to β-amyloid or lipopolysaccharide (LPS) [5,6,7,8,9,10]. LPS in the central nervous system (CNS) has been shown to initiate neurodegenerative diseases such as Alzheimer’s disease through the upregulation of diverse inflammatory genes, including cyclooxygenase (COX)-2, cytosolic phospholipase A_2_, matrix metalloproteinases, and cytokines. Thus, LPS may be a risk factor for the upregulation of inflammatory proteins in neurodegenerative diseases. Although endothelial cells have been claimed to be an important component in brain injury, the implication of these cells in neurodegenerative diseases is still unknown.

Prostaglandins (PGs) and thromboxane A_2_ are formed when the plasma membrane releases the arachidonic acid by the action of phospholipases and is metabolized by the sequential actions of PGG/H synthase or COX. COXs convert the arachidonic acid to PGH_2_, which is enzymatically converted to a series of prostanoids such as PGE_2_, PGF_2_, PGI_2_, and PGD_2_. Among these prostanoids, PGD_2_ can undergo chemical dehydration, losing a molecule of water to form the cyclopentenone prostaglandin PGJ_2_ [11]. PGJ_2_ is further metabolized to produce Δ^12^-PGJ_2_ and 15-deoxy-Δ^12,14^-PGJ_2_ (15d-PGJ_2_). Growing evidence has indicated that the 15d-PGJ_2_ possesses neuroprotection via inducing the Nrf2 pathway in various types of cells [12,13]. In addition, 15d-PGJ_2_ has anti-inflammatory properties such as inhibiting COX-2 expression, preventing IκB degradation, and inhibiting tumor necrosis factor (TNF)-α release [14,15]. However, whether 15d-PGJ_2_ can induce anti-inflammatory proteins such as heme oxygenase (HO)-1 in brain endothelium remains unclear. Thus, we investigated whether 15d-PGJ_2_ can possess the anti-inflammatory effects in a brain damage model created by LPS and dissected the possible mechanisms of its anti-inflammation in mouse brain endothelial (bEnd.3) cells.

The expression of HO-1 is relatively low in the normal brain. However, HO-1 could be markedly upregulated by various disease states in the CNS [16]. Upregulation of HO-1 has been demonstrated to have advantageous effects in many brain injury models, such as ischemia/reperfusion injury [17], traumatic spinal cord injury [18], and traumatic brain injury [19]. HOs are microsomal rate-limiting enzymes in the oxidative degradation of pro-oxidant heme into effective antioxidants, including free iron (ferrous iron), carbon monoxide (CO), and biliverdin which is converted to bilirubin mediated through biliverdin reductase [20]. Biliverdin and bilirubin exert potent antioxidative and anti-nitrosative effects in diverse cell culture models, including endothelial cells [21]. CO exerts potent anti-inflammatory [22] and anti-apoptotic [22] effects and prevents BBB disruption [23] effects in in vitro and in vivo studies. Iron release from HO activity leads to the post-translational release of the translational repression of ferritin. Therefore, ferritin synthesis is increased [24]. Overexpression of ferritin reduced apoptosis in a model of I/R injury after liver transplantation; ferritin confers cytoprotection against oxidative stress and performs an intermediate role in the HO-mediated anti-apoptotic effect [25]. There are three isoforms of HO, including one inducible isoform (HO-1) and two constitutive isoforms (HO-2 and HO-3). All three byproducts of heme are responsible for the cryoprotective properties of HO-1 [21]. Lin et al. revealed that the upregulation of 15d-PGJ_2_ by transferring adenoviral COX-1 (Adv-COX-1) reduced infarct volume in the ischemic cortex and increased HO-1 level in a rat stroke model [26]. Koyani et al. indicated that 15d-PGJ_2_ induces the expression of HO-1 mediated through a reactive oxygen species (ROS) synthesis-activated p38 mitogen-activated protein kinase (MAPK)/Akt/Nrf2-Egr1 cascade in the MG-63 cell line [27]. Another report employing rat vascular smooth muscle cells showed that 15d-PGJ_2_ induces HO-1 upregulation mediated through ROS and the p38 MAPK pathway, leading to Nrf2 nuclear translocation but not peroxisome proliferator-activated receptor (PPAR)-γ [28]. Thus, in the present study, we examine whether 15d-PGJ_2_ can induce HO-1 upregulation through various signaling components in bEnd.3 cells. Our present findings support that 15d-PGJ_2_-stimulated HO-1 upregulation is modulated by a ROS/protein kinase C (PKC)δ/c-Jun N-terminal kinase (JNK)1/2-dependent axis to activate specificity protein 1 (Sp1) and activator protein 1 (AP-1) transcription activities in mouse brain endothelial cells.

## 2. Materials and Methods

### 2.1. Reagents

15d-PGJ_2_, anti-HO-1 polyclonal antibody (ADI-SPA-895), and MitoTEMPO were acquired from Enzo Life Science (Farmingdale, NY, USA). Fetal bovine serum (FBS) and Dulbecco’s modified Eagle’s medium (DMEM)/F-12 were purchased from Invitrogens (Carlsbad, CA, USA). The Western blotting detection system, enhanced chemiluminescence, and Hybond C membrane were from GE Healthcare Biosciences (Buckinghamshire, UK). Ro318220, diphenyleneiodonium chloride (DPI), Rottlerin, and MitoTempo were from Biomol (Plymouth Meeting, PA, USA). Anti-glyceraldehyde-3-phosphate dehydrogenase (GAPDH) antibody (Cat# MCA-1D4) was from EnCor Biotechnology (Gainesville, FL, USA). The bicinchoninic acid protein assay kit was from Pierce (Rockford, IL, USA). Anti-Sp1 (phospho Thr^453^, ab37707) and anti-Sp1(ab227383) antibodies were purchased from Abcam (Cambridge, UK). CM-H_2_DCFDA was from Molecular Probes (Eugene, OR, USA). siRNAs of PKCδ, JNK2, Sp1, and c-Jun, LPS (L2630), the 2,3-bis-(2-methoxy-4-nitro-5-sulfophenyl)-2H-tetrazolium-5-carboxanilide (XTT) assay kit, TRIzol, and other chemicals were from Sigma-Aldrich (St. Louis, MO, USA). Sodium dodecyl sulfate-polyacrylamide gel electrophoresis supplies were from MDBio Inc (Taipei, Taiwan). GenMute™ siRNA Transfection Reagent was purchased from SignaGen Laboratories (Rockville, MD, USA). Anti-lamin A (H-102)(sc-20680), anti-β-actin (C4) (sc-47778), anti-JNK1/2 (E5) (sc-7891), and anti-c-Jun (sc-44) antibodies were purchased from Santa Cruz Biotechnology (Santa Cruz, CA, USA). Anti-phospho-c-Jun (Ser^63^, #2361), anti-phospho-SAPK/JNK(Thr^183^/Tyr^185^), anti-phospho-PKCδ (Thr^505^, #9374), and anti-PKCδ (#9916) were purchased from Cell Signaling Technology (Danvers, MA, USA). Mithramycin A and SP600125 were purchased from Cayman Chemicals (Ann Arbor, MI, USA).

### 2.2. Cell Cultures and Treatment

The bEnd.3 cells, isolated from BALB/c mice brain endothelial cells transformed by a retrovirus vector that expresses polyomavirus middle T antigens, were adopted to study the anti-inflammatory and antioxidative effects on the endothelial cells of the brain. The bEnd.3 cells were purchased from Bioresource Collection and Research Centre (BCRC, Hsinchu, Taiwan) and grew in DMEM/F-12 containing 10% FBS and antibiotics in a humidified 5% CO_2_ atmosphere at 37 °C, as described previously [29]. When grown to confluence, cells were released with 0.05% (*w*/*v*) trypsin. The cell suspension was diluted to a concentration of 2 × 10^5^ cells/mL using DMEM/F-12 containing 10% FBS. The cell suspension was plated onto (1 mL/well) 12-well culture plates and (10 mL/dish) for the analysis of RNA expression, protein, or promoter activity. The culture medium was changed every 4 days.

The cells were identified by an indirect immunofluorescent staining method using a monoclonal antibody of F-VIII to characterize the isolated and cultured bEnd.3 cells and to exclude contamination by fibroblasts and epithelial cells. Over 95% of the cultured cells were reacted positively with an antibody targeting F-VIII.

### 2.3. Protein Preparation and Western Blot Analysis

The bEnd.3 cells at confluence were made quiescent by incubation in serum-free DMEM/F-12 for 24 h. Growth-arrested cells were incubated without or with 15d-PGJ_2_ for the indicated time intervals at 37 °C. When inhibitors were employed, they were administrated 1 h with cells before the exposure to 15d-PGJ_2_.

The cells were rapidly washed with ice-cold phosphate-buffered saline (PBS) and collected in a lysis buffer. The lysates were dissolved in 1.25× sample buffer and heated at 95 °C for 5 min. The denatured proteins were centrifuged at 13,000× *g* for 1 min, as described previously [30]. The mixed samples (15 μL) were subjected to SDS-PAGE using a 10% running gel. Proteins were transferred to a nitrocellulose membrane, and the membrane was incubated sequentially with 5% (*w*/*v*) bovine serum albumin in Tween Tris-buffered saline (TTBS) for 1 h at room temperature. Membranes were incubated with an anti-target protein antibody used at a dilution of 1:1000 in TTBS overnight at 4 °C. Then, membranes were incubated with 1:2000 dilution of an anti-rabbit or anti-mouse horseradish peroxidase-conjugated antibody for 1 h after being washed with TTBS several times. Following incubation, the membranes were washed extensively with TTBS. The immunoreactive bands were detected using enhanced chemiluminescence reagents and captured by a UVP BioSpectrum 500 Imaging System (Upland, CA, USA). To conduct the image densitometry analysis, UN-SCAN-IT gel software (Orem, UT, USA) was used.

### 2.4. Transient Transfection with siRNAs in bEnd.3 Cells

Mouse siRNAs of SMARTpool RNA duplexes corresponding to NOX4 (EMU023701, NM_015760), Sp1 (EMU061231; NM_013672), and scrambled control (negative control type 1) siRNA were from Sigma-Aldrich (St. Louis, MO, USA). The following siRNA duplexes were used: PKCδsiRNA (NM_011103.3) sense, 5′-CCAUGUAUCCUGAGUGGAATT-3′ and antisense, 5′-UUCCACUCAGGAUACAUGGTT-3′. c-Jun siRNA (sc-29224, NM_010591.2) and JNK2 siRNA (sc-39102, NM_001163671.1) were from Santa Cruz Biotechnology (Santa Cruz, CA, USA). Briefly, transient transfection of siRNAs was performed by using Genmute reagent and Opti-MEM, as described previously [30]. The transfection complex (Genmute reagent 2.5 μL, Opti-MEM 100 μL, and siRNA 100 nM) was directly administrated to the cells and incubated for 5 h. Transfection complex medium was replaced with DMEM/F-12 medium containing 10% FBS overnight. Then, the cells were changed to a serum-free medium for 24 h.

### 2.5. Quantitative Real-Time PCR Analysis

Total RNA was extracted from bEnd.3 cells after 10 μM 15d-PGJ_2_ stimulation for various time points in culture dishes (10-cm) using 1 mL TRIzol reagent, as described previously [29]. The levels of RNA were spectrophotometrically measured at 260 nm. mRNA was reverse-transcribed into cDNA and determined by real-time RT-PCR, as described previously [30]. Real-time PCR was performed using Kapa Probe Fast qPCR Kit Master Mix (2X) Universal (KK4705; KAPA Biosystems, Wilmington, MA, USA), a StepOnePlusTM real-time qPCR system (ThermoScientific-Applied Biosystems, Foster City, CA, USA), and primers specific for HO-1 (NM_010442.2): 5′-CACGCATATACCCGCTACCT-3′ (sense); 5′-TCT GTCACCCTGTGCTTGAC-3′ (antisense) and for interleukin-6 (IL-6; NM_001314054.1): 5′-ACAACCACGGCCTTCCCTACTT-3′ (sense), 5′-CACGATTTCCCAGAGAACATGTG-3′ (antisense). The levels of mRNA expression were analyzed by normalizing to GAPDH expression with primers as the following: GAPDH (XM_036165840.1): 5′-GGGCTGCC CAGAACATCAT-3′(sense); 5′-CAGATCCA CGACGGACACATT-3′(antisense). Relative gene expression was calculated by the ΔΔCt method, where Ct meant the threshold cycle. All experiments were carried out in triplicate.

### 2.6. Measurement of Intracellular ROS Accumulation

This method is based on detecting fluorescent 2′-7′dichlorofluorescein (DCF), oxidatively converted from non-fluorescent 2′,7′-dichlorodihydrofluorescein diacetate (H_2_DCF-DA) by H_2_O_2_, as described previously [30]. The intracellular H_2_O_2_ levels were determined by measuring the fluorescent intensity of DCF. For this intention, cells were washed with warm PBS and incubated in PBS containing 10 μM H_2_DCF-DA for 30 min at 37 °C. Successively, PBS containing DCFH-DA was discarded and changed to a fresh medium. Cells were treated with 15d-PGJ_2_ in the absence or presence of inhibitors of NOX or a ROS scavenger. Cells were washed twice with PBS, and then fluorescent intensity was measured. The fluorescent intensity was detected using a fluorescent microplate reader (SynergyH1 Hybird Reader, BioTek, Winooski, VT, USA) at 495/529 nm and FACSCalibur equipped with CellQuest software (BD Biosciences, San Jose, CA, USA).

### 2.7. NOX Activity Assay

The cells were gently scraped and centrifuged at 400× *g* for 10 min at 4 °C after exposure to 10 μM 15d-PGJ_2_ for the indicated time intervals. The cell pellet kept on ice was resuspended with ice-cold PBS (35 μL). To a final 200 μL volume of pre-warmed (37 °C) PBS containing either lucigenin (20 μM) or NADPH (1 μM), 5 μL of cell suspension (2 × 10^4^ cells) was added to initiate the reaction, followed by immediate measurement of chemiluminescence in a luminometer (SynergyH1 Hybird Reader, BioTek, Winooski, VT, USA), as described previously [31].

### 2.8. Cytoplasmic and Nuclear Protein Extraction

The assay was performed by the method with modifications, as described previously [31]. bEnd.3 cells were seeded onto culture dishes (10-cm). The cells were starved for 24 h in DMEM/F-12 medium without FBS when they reached 90% confluence. After incubation with 10 μM 15d-PGJ_2_ for the time intervals, the cells were washed once with ice-cold PBS and scraped into a 1.5 mL tube with 1 mL of PBS added to each dish. Cells were collected by centrifuge at 8000 rpm for 5 min. The pellets were suspended with 300 μL Cytoplasmic Extraction Reagent I (CREI). The suspension was broken by syringes. After being put on ice for 30 min, the lysates were centrifuged at 8000 rpm for 10 min. The pellet was collected as the nucleus fraction and the supernatant as a cytosol fraction. The pellets were resuspended and then sonicated for 5 s twice using a sonicator (Misonix, Farmingdale, NY, USA). The protein concentration of each sample was measured. Samples from these fractions (200 μL protein) were denatured and subjected to SDS-PAGE using a 12% (*w*/*v*) running gel. The levels of translocation were identified and quantified by Western blot analysis.

### 2.9. Chromatin Immunoprecipitation Assay (ChIP)

The assay was performed by the method with modifications, as described previously [30]. In short, bEnd.3 cells stimulated with 10 μM 15d-PGJ_2_ for the indicated time points were cross-linked with 1% formaldehyde for 10 min at 37 °C and washed thrice with ice-cold PBS containing 1% aprotinin and 1 mM phenylmethyl-sulfonyl fluoride. The purified DNA was subjected to amplification by real-time PCR using the primers specific for the region (−460 to −2; NC_000074.7) containing a putative AP-1 binding site present in the mHO-1 promoter region (sense primer: 5′-GGACGCGGAGGAGCAGGGGCTAGCAT-3′; antisense primer: 5′-CACGTCCGCTCTCCTTGCCAGGACT-3′) with the product size of 238 bp and Sp1 binding site present in the mHO-1 promoter region (sense primer: 5′-AGACTTGCCAGAGTCATATGATTTATCCCCTTAC-3′; antisense primer: 5′-GCTCGAGACGGCTCTGCGCGGGCAGGCTCCAC-3′) with the product size of 224 bp. Real-time PCR was carried out using Luna Universal qPCR Master Mix (M3003; New England BioLabs, Ipswich, MA, USA) on a StepOnePlus™ real-time PCR system (Applied Biosystems, Foster City, CA, USA).

### 2.10. Preparation of Recombinant Adenovirus

A recombinant adenovirus containing human HO-1 (AdvHO-1) was kindly provided by Dr. L.Y. Chau (Institute of Biomedical Sciences, Academia Sinica, Taipei, Taiwan). Recombinant adenovirus was generated by homologous recombination and amplified in 293 cells. Large scales of viral vectors were purified by CsCl ultracentrifugation and stored in 10% (*v*/*v*) glycerol, 1 mmol/L MgCl_2_, and 10 mmol/L Tris-HCl (pH 7.4) at −80 °C until used for experiments. Virus titers were measured on a 293-cell monolayer by a plaque assay. The recombinant adenovirus was diluted with DMEM/F12 medium and added directly to the cells based on the indicated multiplicity of infection (MOI = 20, 30, and 40), as described previously [32]. The cells were incubated with LPS for the indicated time intervals after 24 h of infection. Cell lysates and the media were analyzed by Western blotting and ELISA kits, respectively.

### 2.11. Measurement of IL-6 Generation

The levels of IL-6 released into the media of bEnd.3 cell cultures were detected using an ELISA kit (R&D System, Minneapolis, MN, USA) according to the manufacturer’s instructions (https://www.rndsystems.com/products/mouse-il-6-quantikine-elisa-kit_m6000b; Accessed date: 3 April 2022).

### 2.12. Statistical Analysis of Data

GraphPad Prism Program 6.0 software (GraphPad, San Diego, CA, USA) was used to perform statistical analysis. We used one-way ANOVA followed by Dunnett’s post hoc test when comparing more than two groups of data, as previously described [30]. *p*-values of 0.01 were statistically significant. Post hoc tests were analyzed only if F achieved *p* < 0.01 and there was no variance inhomogeneity. Error bars were omitted when they fell within the dimensions of the symbols. All the data were expressed as the mean ± SEM, three individual experiments (*n* = 3).

## 3. Results

### 3.1. 15d-PGJ_2_ Induces Expression of HO-1 mRNA and Protein in bEnd.3 Cells

**Figure 1:**
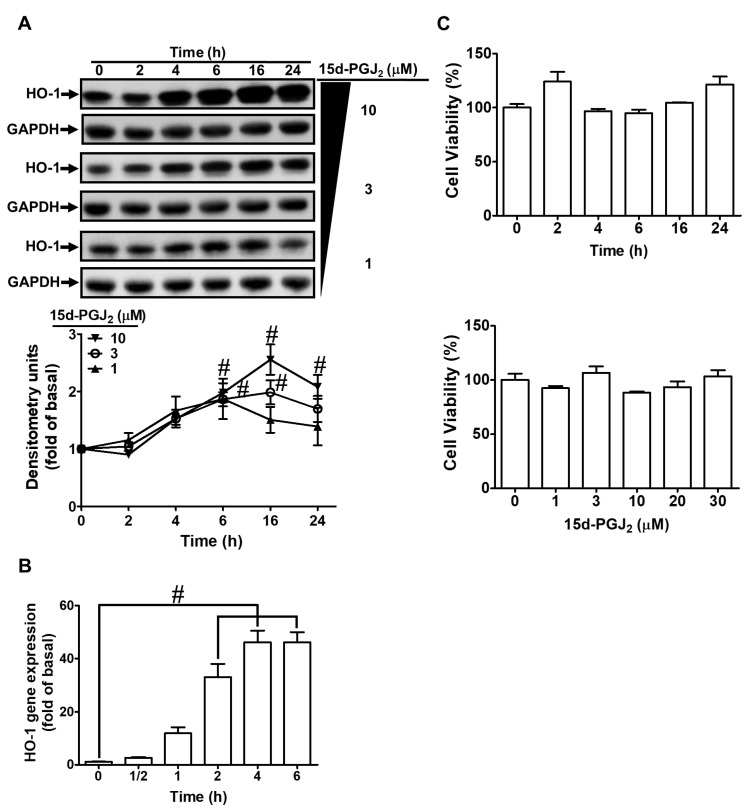
15d-PGJ_2_ induces gene and protein expression of HO-1 in bEnd.3 cells. (**A**) The cells were incubated with 15d-PGJ_2_ (1, 3, 10 μM) for 0, 2, 4, 6, 16, and 24 h. The cell lysates were examined by Western blotting using the polyclonal antibody of HO-1 or GADPH (serving as a loading control). (**B**) The cells were stimulated with 10 μM 15d-PGJ_2_ for 0.5, 1, 2, 4, and 6 h. The HO-1 gene expression was analyzed by real-time PCR. (**C**) The cells were incubated with 10 μM of 15d-PGJ_2_ for 2, 4, 6, 16, and 24 h (upper panel) and with various doses (1, 3, 10, 20, and 30 μM; lower panel) for 24 h, and then cell viability was analyzed by an XTT assay. Data are expressed as the mean ± S.E.M. of three independent experiments. ^#^ *p* < 0.01, as compared with the vehicle alone or basal level. Abbreviations: 15d-PGJ_2_: 15-deoxy-Δ^12,14^-prostaglandin J_2_; HO-1: heme oxygenase-1.

bEnd.3 cells were incubated with 1, 3, and 10 μM 15d-PGJ_2_ for the indicated time points to examine the effect of 15d-PGJ_2_ on the expression of HO-1. As shown in Figure 1A, 15d-PGJ_2_ produced a time- and concentration-dependent expression of HO-1 protein assessed by Western blot. The response reached a significant rise within 6 h, with a maximal level within 16 h, and slightly declined within 24 h. The mRNA expression of HO-1 was also time-dependently induced by15d-PGJ_2_ with a maximal response within 6 h, evaluated by real-time PCR (Figure 1B) during the period of observations. Additionally, the findings of the XTT assay (Figure 1C) indicated that there was no cellular toxicity under 30 μM 15d-PGJ_2_ treatment within 24 h. These results indicated that HO-1 expression could be induced by 15d-PGJ_2_ in bEnd.3 cells. The concentration of 15d-PGJ_2_ 10 μM used in this study was based on previous studies and the inhibitory efficacies of inhibitors [28,33].

### 3.2. 15d-PGJ_2_ Induces HO-1 Expression via Mitochondria- and NOX-Generated ROS

**Figure 2:**
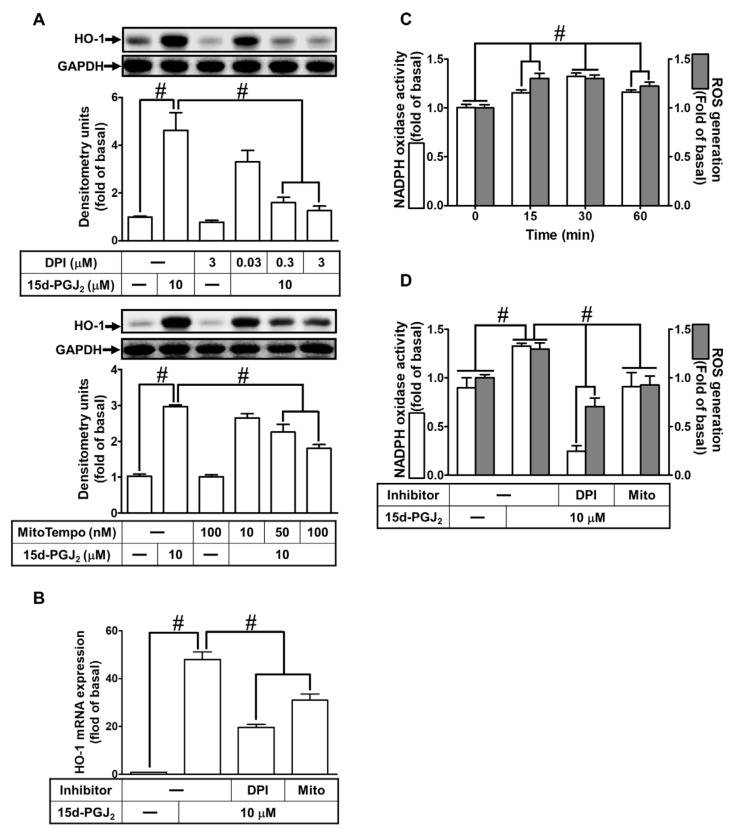
15d-PGJ_2_ induced expression of HO-1 in bEnd.3 cells via NOX activation and mitochondria-generated ROS. (**A**) The cells were pretreated with the indicated doses of DPI or MitoTempo for 1 h before incubation with 10 μM 15d-PGJ_2_ for 6 h. To determine the HO-1 protein expression, the cell lysates were analyzed by Western blot with GAPDH as an internal control. (**B**) The cells were pretreated by 3 μM DPI or 100 nM MitoTempo for 1 h before incubation with 15d-PGJ_2_ (10 μM) for 4 h. The HO-1 gene expression was analyzed by real-time PCR. (**C**) The cells were incubated with 10 μM 15d-PGJ_2_ for 15, 30, and 60 min. (**D**) Cells were pretreated with MitoTempo (100 nM) or DPI (3 μM) for 1 h and then stimulated with 15d-PGJ_2_ (10 μM) for 30 min. (**C**,**D**) The activity of NOX and ROS generation were measured by NOX activity assay and H_2_DCF-DA assay, respectively. Data are expressed as the mean ± S.E.M. of three independent experiments. ^#^ *p* < 0.01, as compared with the basal level or the cells treated with 15d-PGJ_2_ alone. Abbreviations: DPI: diphenyleneiodonium chloride; Mito: MitoTempo; NOX: NADPH oxidase; ROS: reactive oxygen species.

ROS could be produced by an enzymatic source such as NOX under various pathological conditions. NOX/ROS has been revealed to induce HO-1 expression, which possesses anti-inflammatory and cytoprotecting functions [34]. Thus, we investigated whether, in bEnd.3 cells, NOX activity and ROS production contribute to 15d-PGJ_2_-induced HO-1 expression. Cells were pretreated with the indicated dosages of DPI (an inhibitor of NOX) or MitoTempo (a mitochondria-targeted antioxidant agent) for 1 h before exposure to 15d-PGJ_2_ (10 μM) for 6 h. As presented in Figure 2A, preprocessing cells with DPI or MitoTempo significantly diminished the protein expression of HO-1 as compared with those of the basal levels. Similarly, 15d-PGJ_2_-induced mRNA expression of HO-1 was also attenuated by these two inhibitors (Figure 2B). Further, our data unveiled the participation of NOX-derived ROS generation in 15d-PGJ_2_-induced HO-1 upregulation in bEnd.3 cells by measuring NOX activity and generation of ROS. As displayed in Figure 2C, 15d-PGJ_2_, in a time-dependent manner, promoted NOX activity and ROS production with a maximal response within 30 min during the observation periods. Moreover, pretreatment with either DPI or MitoTempo attenuated 15d-PGJ_2_-stimulated both NOX activity and ROS generation (Figure 2D). These findings indicated that 15d-PGJ_2_ could stimulate NOX activity, leading to ROS production in these cells. Thus, in bEnd.3 cell, NOX-derived generation of ROS plays a critical role in 15d-PGJ_2_-induced HO-1 expression.

### 3.3. Involvement of PKCδ in 15d-PGJ_2_-Induced Expression of HO-1

**Figure 3:**
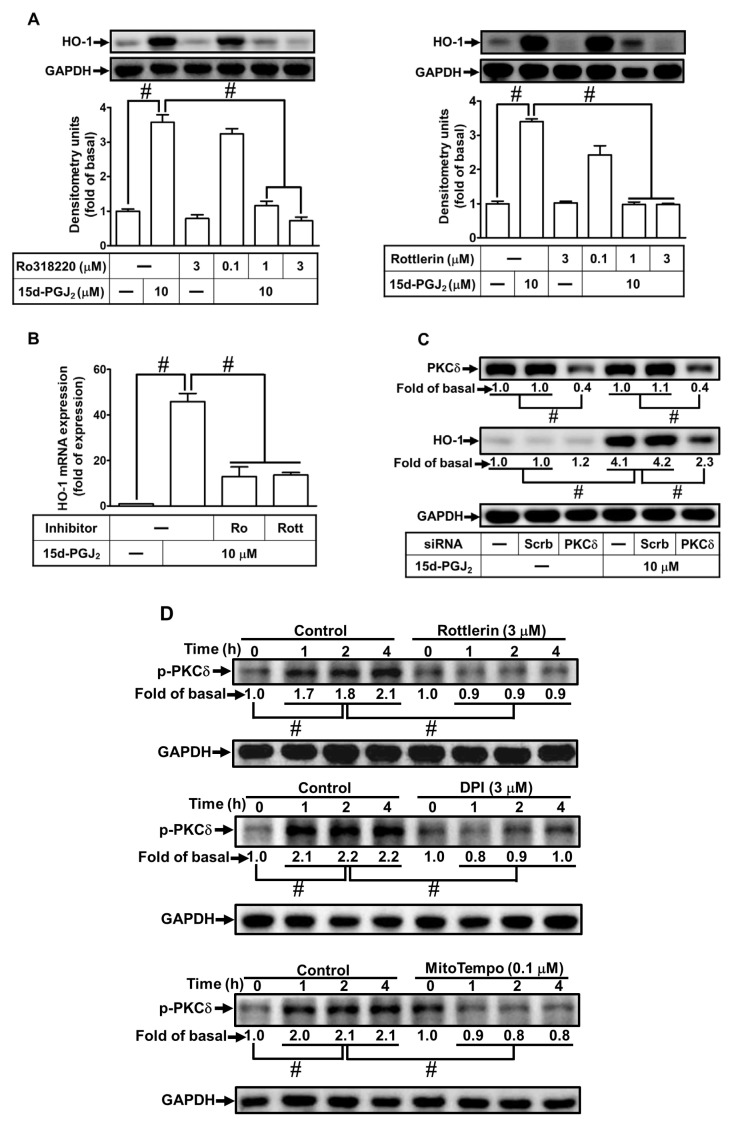
Involvement of PKCδ in HO-1 upregulated by 15d-PGJ_2_ in bEnd.3 cells. (**A**) The indicated doses of Ro318220 or Rottlerin pretreated cells for 1 h before cells were incubated with 15d-PGJ_2_ (10 μM) for 6 h. To determine the HO-1 expression, the cell lysates were assessed by Western blot with GAPDH as an internal control. (**B**) The cells were pretreated with Rottlerin (3 μM) or Ro318220 (3 μM) for 1 h and then incubated with 10 μM 15d-PGJ_2_ for 4 h. mRNA levels of HO-1 were quantified by real-time PCR. (**C**) The cells were transfected with scrambled or PKCδ siRNA and then challenged with 15d-PGJ_2_ (10 μM) for 6 h. The PKCδ and HO-1 protein expression were assessed by Western blot with GAPDH as an internal control. (**D**) The cells were preprocessed with Rottlerin (3 μM), DPI (3 μM), or MitoTempo (100 nM) for 1 h and then challenged by 15d-PGJ_2_ (10 μM) for 1, 2, and 4 h. The phosphorylation of PKCδ was assessed by Western blot with GAPDH as an internal control. Data are expressed as the mean ± S.E.M. of three independent experiments. ^#^ *p* < 0.01, as compared with the basal level or 15d-PGJ_2_ treatment alone. Abbreviations: Scrb: scrambled; Ro: Ro318220; Rott: Rottlerin.

**Figure 4:**
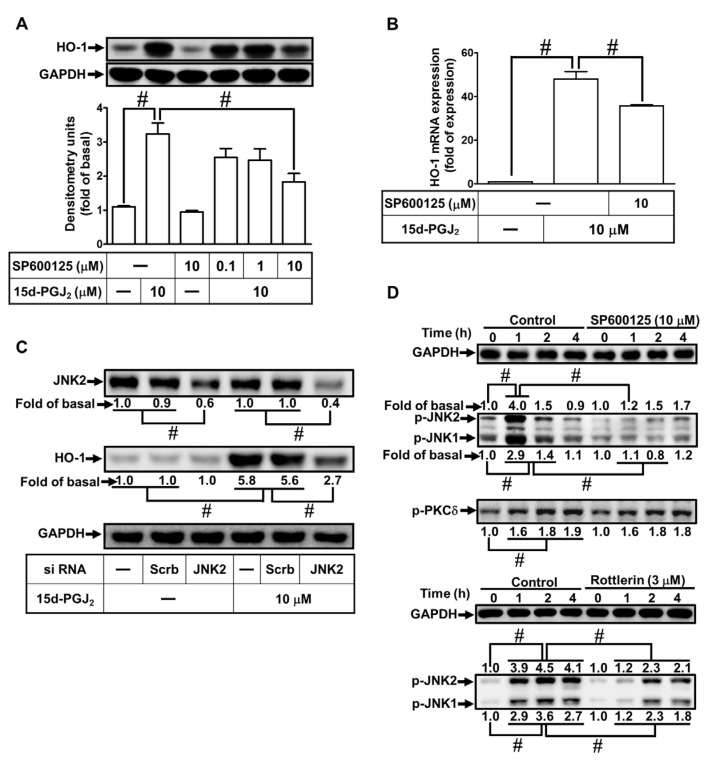
Involvement of JNK1/2 in HO-1 expression stimulated by 15d-PGJ_2_ in bEnd.3 cells. (**A**) The cells were pretreated with the indicated doses of SP600125 for 1 h before incubation with 15d-PGJ_2_ (10 μM) for 6 h. To determine the HO-1 protein expression, the cell lysates were examined by Western blot with GAPDH as an internal control. (**B**) The cells were pretreated with SP600125 (10 μM) for 1 h and then treated with 15d-PGJ_2_ (10 μM) for 4 h. The mRNA levels of HO-1 were quantified by real-time PCR. (**C**) The cells were transfected with JNK2 siRNA and then challenged by 15d-PGJ_2_ (10 μM) for 6 h. The protein levels of JNK2 and HO-1 were examined by Western blot with GAPDH as an internal control. (**D**) The cells pretreated with SP600125 (10 μM) or Rottlerin (3 μM) for 1 h were stimulated by 15d-PGJ_2_ (10 μM) for 1, 2, and 4 h. The phosphorylation of JNK1/2 or PKCδ was determined by Western blot with GAPDH as an internal control. Data are expressed as the mean ± S.E.M. of three independent experiments. *^#^ p* < 0.01, as compared with the basal level or treatment with 15d-PGJ_2_ alone. Abbreviations: JNK: c-Jun N-terminal kinase.

Previous studies have uncovered that PKCs can upregulate HO-1 expression [35]. PKCs activated by 15d-PGJ_2_ have important effects on cellular functions [36]. The selective inhibitor of PKCδ (Rottlerin) and a non-selective PKC inhibitor (Ro318220) were adopted to address the possible role of PKC activation in 15d-PGJ_2_-induced HO-1 expression. bEnd.3 cells were pretreated, each above the inhibitor, for 1 h and then stimulated with 15d-PGJ_2_ (10 μM) for 6 h. As exhibited in Figure 3A, pretreating cells with Rottlerin or Ro318220 dose-dependently attenuated 15d-PGJ_2_-induced HO-1 expression. Additionally, pretreating cells with Ro318220 or Rottlerin also attenuated 15d-PGJ_2_-promoted HO-1 mRNA levels (Figure 3B). We used PKCδ siRNA transfection to ensure the role of PKCδ in 15d-PGJ_2_-induced HO-1 expression in bEnd.3 cells. Consistent with the results obtained with Rottlerin, the knockdown of PKCδ by PKCδ siRNA transfection blocked the 15d-PGJ_2_-stimulated HO-1 protein level (Figure 3C). Further, we investigated whether phosphorylation of PKCδ was involved in 15d-PGJ_2_-mediated responses. As shown in Figure 3D, 15d-PGJ_2_ can stimulate phosphorylation of PKCδ within the period observed, which was inhibited by respective pretreatment with Rottlerin, DPI, or MitoTempo. Pretreatment with SP600125 did not significantly inhibit PKCδ phosphorylation induced by 15d-PGJ_2_ (Figure 4D). These results suggested that in bEnd.3 cells, PKCδ, as a downstream signaling molecule of the NOX/ROS cascade, is essential for the HO-1 expression induced by 15d-PGJ_2_.

### 3.4. 15d-PGJ_2_ Enhances Expression of HO-1 via JNK1/2

Stimulation of JNK1/2 in various types of cells represents one of the main regulatory mechanisms induced by 15d-PGJ_2_ [37,38]. To explore whether, in bEnd.3 cells, JNK1/2 is engaged in 15d-PGJ_2_-induced HO-1 expression, the cells were preprocessed with the indicated dosage of SP600125 (an inhibitor of JNK1/2) for 1 h before incubation with 10 μM 15d-PGJ_2_ for 6 h. As presented in Figure 4A, pretreating cells with SP600125 significantly reduced HO-1 expression induced by 15d-PGJ_2_. In addition, 15d-PGJ_2_-induced mRNA expression of HO-1 was attenuated by SP600125 pretreatment (Figure 4B). To ascertain that JNK1/2 participates in the expression of HO-1, the bEnd.3 cells were transfected with JNK2 siRNA. As shown in Figure 4C, downregulation of JNK2 attenuated the 15d-PGJ_2_-stimulated HO-1 expression. Further, we investigate whether phosphorylation of JNK1/2 was required for 15d-PGJ_2_-mediated responses. The data in Figure 4D showed that 15d-PGJ_2_ could stimulate JNK1/2 phosphorylation, which was blocked by either SP600125 or Rottlerin pretreatment. These findings suggest that in bEnd.3 cells, 15d-PGJ_2_-stimulated HO-1 upregulation can be mediated through a PKCδ-dependent JNK1/2 pathway.

### 3.5. Sp1 Is Involved in 15d-PGJ_2_ Induced HO-1 Upregulation

**Figure 5:**
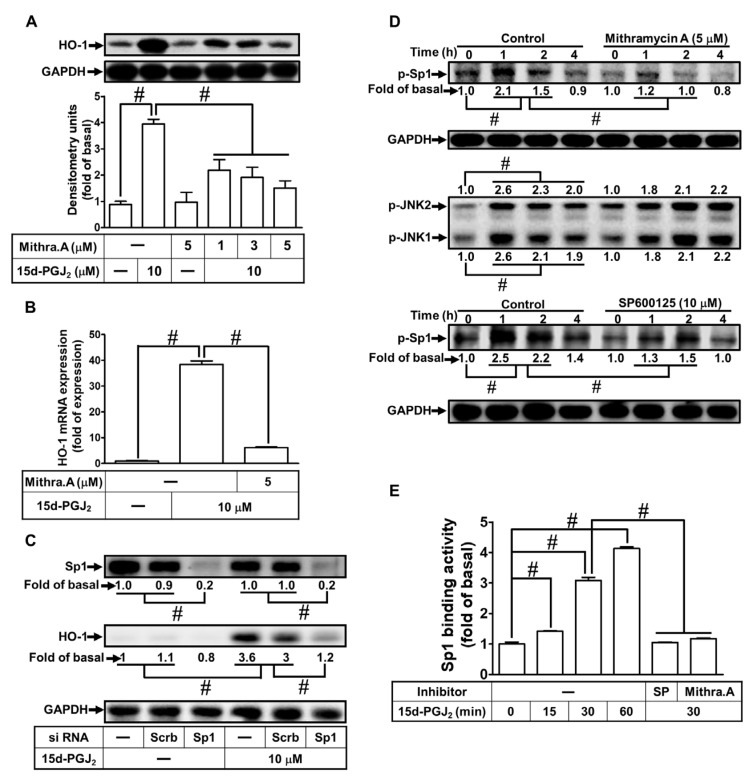
Involvement of transcription factor Sp1 in HO-1 expression stimulated by 15d-PGJ_2_ in bEnd.3 cells. (**A**) The cells were preprocessed with Mithramycin A (1, 3, and 5 μM) for 1 h and then challenged with 15d-PGJ_2_ (10 μM) for 6 h. The HO-1 protein expression was determined by Western blot with GAPDH as an internal control. (**B**) Mithramycin A (5 μM) pretreated the bEnd.3 cells for 1 h, which were challenged with 15d-PGJ_2_ (10 μM) for 4 h. The mRNA expression of HO-1 was analyzed by real-time PCR. (**C**) The bEnd.3 cells were transfected with scrambled or Sp1 siRNA and then challenged with 15d-PGJ_2_ (10 μM) for 4 h. The levels of HO-1 and Sp1 protein expression were determined by Western blot with GAPDH as an internal control. (**D**) bEnd.3 cells were preprocessed with Mithramycin A (5 μM) or SP600125 (10 μM) for 1 h and then administrated with 15d-PGJ_2_ (10 μM) for 1, 2, and 4 h. The phosphorylation levels of Sp1 and JNK1/2 were determined by Western blot with GAPDH as an internal control. (**E**) bEnd.3 cells were stimulated with 15d-PGJ_2_ for 15, 30, and 60 min alone or 30 min while cells were pretreated with Mithramycin A (5 μM) or SP600125 (10 μM) for 1 h. The binding of Sp1 to the promoter region of HO-1 was determined with a ChIP assay. Data are expressed as the mean ± S.E.M. of three independent experiments. *^#^ p* < 0.01, as compared with the basal level or treatment with 15d-PGJ_2_ alone. Abbreviations: Mithra. A: Mithramycin A; SP: SP600125; Sp1: specificity protein 1.

Previous studies have unveiled that Sp1 in various types of cells is an important transcription factor that is involved in the expression of HO-1 [39]. To evaluate whether Sp1 is involved in the HO-1 expression, the bEnd.3 cells were preprocessed with Mithramycin A (1, 3, and 5 μM) for 1 h and then stimulated with 15d-PGJ_2_ for 6 h. As demonstrated in Figure 5A, 15d-PGJ_2_-induced protein levels of HO-1 expression were dose-dependently reduced by Mithramycin A. In addition, 15d-PGJ_2_-induced gene expression of HO-1 at 4 h was attenuated by pretreatment with 5 μM Mithramycin A for 1 h (Figure 5B). We further adopted scrambled or Sp1 siRNA transfection to certify the function of Sp1 in the 15d-PGJ_2_-induced expression of HO-1 in bEnd.3 cells. The results in Figure 5C showed that knockdown of Sp1 protein attenuated the 15d-PGJ_2_-induced HO-1 induction, consistent with data obtained with Mithramycin A. Moreover, to investigate whether phosphorylation of Sp1 participated in 15d-PGJ_2_-mediated responses, as demonstrated in Figure 5D, 15d-PGJ_2_-stimulated phosphorylation of Sp1 reached a maximal increase within 1–2 h, which was inhibited by pretreating cells with Mithramycin A (5 μM) or SP600125 (10 μM). Pretreatment with Mithramycin A did not significantly block the phosphorylation of JNK1/2 stimulated by 15d-PGJ_2_. According to these data, we demonstrated that the phosphorylation of JNK1/2 mediated Sp1 activation, which, in turn, led to HO-1 upregulation in bEnd.3 cells stimulated with 15d-PGJ_2_. To verify the binding activity of Sp1 with the HO-1 promoter, the cells were treated by 15d-PGJ_2_ (10 μM) for 15, 30, and 60 min, and cell lysates were evaluated by a ChIP assay using an Sp1 antibody for immunoprecipitation and primers of Sp1 promoter for amplification DNA. The data proved that 15d-PGJ_2_-enhanced binding of Sp1 with the HO-1 promoter was significantly increased within 15–60 min, which was attenuated to the basal level by pretreatment with either SP600125 or Mithramycin A (Figure 5E). These findings suggest that in bEnd.3 cells, Sp1 possesses a key effect in regulating HO-1 expression through JNK1/2-dependent Sp1 activation triggered by 15d-PGJ_2_.

### 3.6. c-Jun Is Involved in 15d-PGJ_2_ Stimulated Expression of HO-1

**Figure 6:**
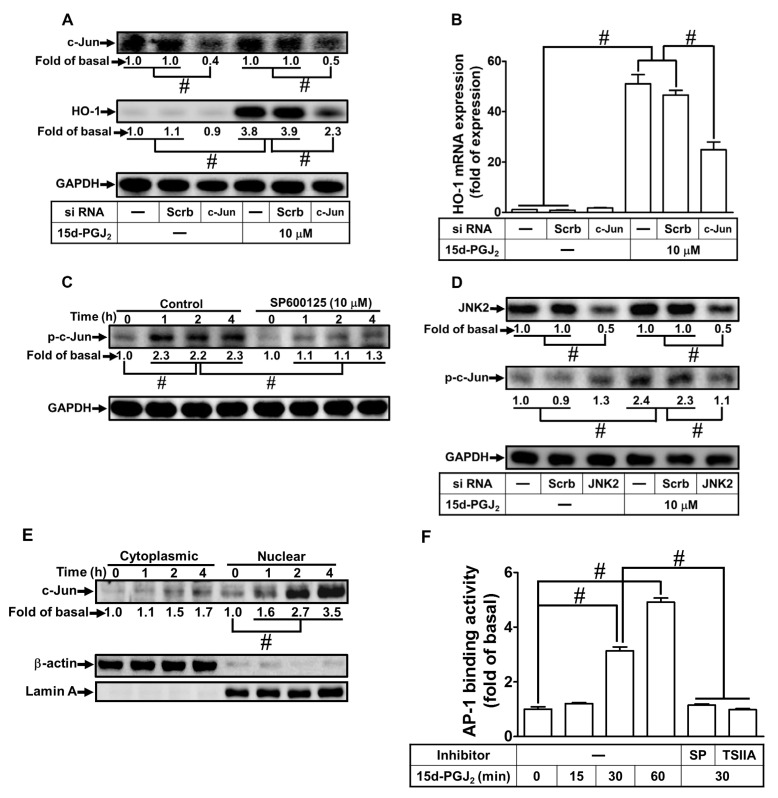
Involvement of transcription factor c-Jun in the expression of HO-1 induced by 15d-PGJ_2_ in bEnd.3 cells. (**A**,**B**) The cells were transfected with scrambled or c-Jun siRNA and then challenged with 15d-PGJ_2_ (10 μM) for 6 h (**A**) or 4 h (**B**). (**A**) The levels of HO-1 protein expression were analyzed by Western blot with GAPDH as an internal control. (**B**) The HO-1 mRNA expression was analyzed by real-time PCR. (**C**) bEnd.3 cells were pretreated with SP600125 (10 μM) for 1 h and then administrated with 15d-PGJ_2_ (10 μM) for 1, 2, and 4 h. The phosphorylation of c-Jun was determined by Western blot with GAPDH as an internal control. (**D**) The bEnd.3 cells were transfected with scrambled or JNK2 siRNA and then challenged with 15d-PGJ_2_ (10 μM) for 4 h. The phosphorylation of c-Jun was examined by Western blot with GAPDH as an internal control. (**E**) bEnd.3 cells were stimulated with 15d-PGJ_2_ for 1, 2, and 4 h. The cells were harvested, and cytosolic and nuclear fractions were separated by centrifugation. The nuclear and cytoplasmic fractions were analyzed by Western blot to evaluate the c-Jun expression, with GAPDH (cytosol) and Lamin A (nucleus) as internal controls. (**F**) bEnd.3 cells were stimulated with 15d-PGJ_2_ for 15, 30, and 60 min alone or 30 min while cells were pretreated with Tanshinone IIA (3 μM) or SP600125 (10 μM) for 1 h. The binding of c-Jun to the promoter region of HO-1 was determined by a ChIP assay. Data are expressed as the mean ± S.E.M. of three independent experiments. *^#^ p* < 0.01, as compared with the basal level or the cells treated with 15d-PGJ_2_ alone. Abbreviations: AP-1: activator protein 1; TSIIA: Tanshinone IIA.

c-Jun has been revealed to regulate HO-1 induction as an important transcription factor. The bEnd.3 cells were transfected with c-Jun siRNA to knock down the level of total c-Jun protein expression and to evaluate whether c-Jun is involved in HO-1 induction. We observed that downregulation of c-Jun protein using c-Jun siRNA attenuated the 15d-PGJ_2_-upregulated protein level (Figure 6A) and gene expression (Figure 6B) of HO-1. Furthermore, to investigate whether c-Jun phosphorylation is involved in 15d-PGJ_2_-mediated effects, as shown in Figure 6C, 15d-PGJ_2_-stimulated c-Jun phosphorylation reached a maximal level within 1-4 h, which was inhibited by pretreating cells with SP600125. To examine whether the phosphorylation of c-Jun was dependent on JNK1/2 activity, the cells were transfected with JNK2 siRNA and then challenged with 10 μM 15d-PGJ_2_ for 4 h. As demonstrated in Figure 6D, downregulation of JNK2 can attenuate the 15d-PGJ_2_-stimulated phosphorylation of c-Jun. According to these data, we demonstrated that JNK1/2 mediated c-Jun activation, which, in turn, led to the expression of HO-1 in bEnd.3 cells stimulated by 15d-PGJ_2_. Moreover, our data also unveiled that 15d-PGJ_2_ stimulated c-Jun translocation into nuclear fractions of bEnd.3 cells (Figure 6E). To verify the binding activity of c-Jun with the HO-1 promoter, 15d-PGJ_2_ (10 μM) treated cells for 15, 30, and 60 min, and the cell lysates were evaluated by a ChIP assay using a c-Jun antibody for immunoprecipitation and primers of the AP-1 promoter. The data demonstrated that the 15d-PGJ_2_-stimulated binding of c-Jun with the HO-1 promoter was significantly augmented within 30–60 min, which was reduced by pretreatment with Tanshinone IIA or SP600125 (Figure 6F). These results indicate that c-Jun has a significant role in regulating the expression of 15d-PGJ_2_-induced HO-1 mediated through the JNK1/2-dependent activation of the AP-1 cascade in bEnd.3 cells.

### 3.7. 15d-PGJ_2_-Induced HO-1 Upregulation Attenuates LPS-Stimulated IL-6 Secretion

**Figure 7:**
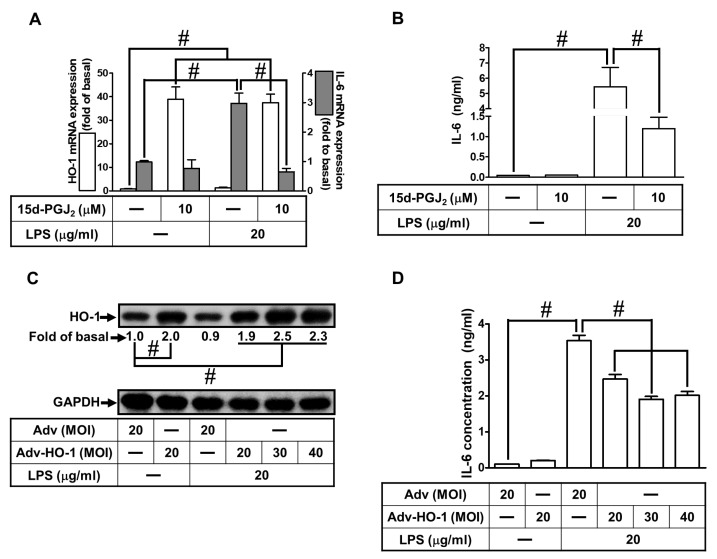
HO-1 upregulation by 15d-PGJ_2_ attenuates LPS-stimulated IL-6 secretion in bEnd.3 cells. (**A**,**B**) The cells were pretreated with 10 μM 15d-PGJ_2_ for 1 h and then challenged with 20 μg/mL LPS for 4 h. (**A**) The cell lysates were collected to analyze the mRNA expression of IL-6 and HO-1 by real-time PCR. (**B**) The media were collected to measure the secretion of IL-6 by an ELISA kit. (**C**,**D**) Cells were infected with the vector of adenovirus or adenovirus-HO-1 at the indicated MOI (20, 30, and 40) for 48 h and then challenged with or without 20 μg/mL LPS for 6 h. (**C**) The cell lysates were examined for the protein expression of HO-1 by Western blot, with GAPDH as an internal control. (**D**) The media were collected to measure the secretion of IL-6 by an ELISA kit. Data are summarized and expressed as mean ± S.E.M. for four individual experiments. *^#^ p* < 0.01, as compared with the basal level or the cells treated with 15d-PGJ_2_ alone. Abbreviations: Adv: adenovirus; IL-6: interleukin-6; LPS: lipopolysaccharide; MOI: multiplicity of infection.

IL-6 is recognized as a crucial inflammatory mediator in brain diseases. To determine whether upregulation of HO-1 can downregulate IL-6 expression and secretion, we pretreated bEnd.3 cells with 10 μM 15d-PGJ_2_ for 1 h and then challenged them with LPS (20 μg/mL ) for 6 h and observed the levels of HO-1 and IL-6 mRNA. The data in Figure 7A show that 15d-PGJ_2_ enhanced the mRNA expression of HO-1 (open bars) and reduced LPS-stimulated IL-6 mRNA expression (solid bars) in bEnd.3 cells. Next, the levels of IL-6 secretion into media were measured using an ELISA kit. We verified that in bEnd.3 cells, LPS-stimulated secretion of IL-6 was also attenuated by pretreatment with 15d-PGJ_2_ (Figure 7B). In addition, we determined whether 15d-PGJ_2_-attenuated IL-6 secretion was mediated through the upregulation of HO-1. bEnd.3 cells were infected with the vector of adenovirus or adenovirus-HO-1 in various MOIs. As demonstrated in Figure 7C, the protein levels of HO-1 were gradually increased by infecting cells with adv-HO-1 at the indicated doses (20, 30, and 40 MOI) but not by LPS. bEnd.3 cells were infected with the vector of adenovirus or adenovirus-HO-1 (20, 30, and 40 MOI) and then were stimulated with LPS (20 μg/mL). As demonstrated in Figure 7D, upregulation of HO-1 by infection with adenovirus-HO-1 (20, 30, and 40 MOI) attenuated IL-6 secretion induced by LPS compared with infection with the vector of adenovirus (20 MOI). These findings indicated that 15d-PGJ_2_-induced expression of HO-1 can exert anti-inflammatory effects on LPS-triggered expression and secretion of IL-6 in bEnd.3 cells.

## 4. Discussion

**Figure 8:**
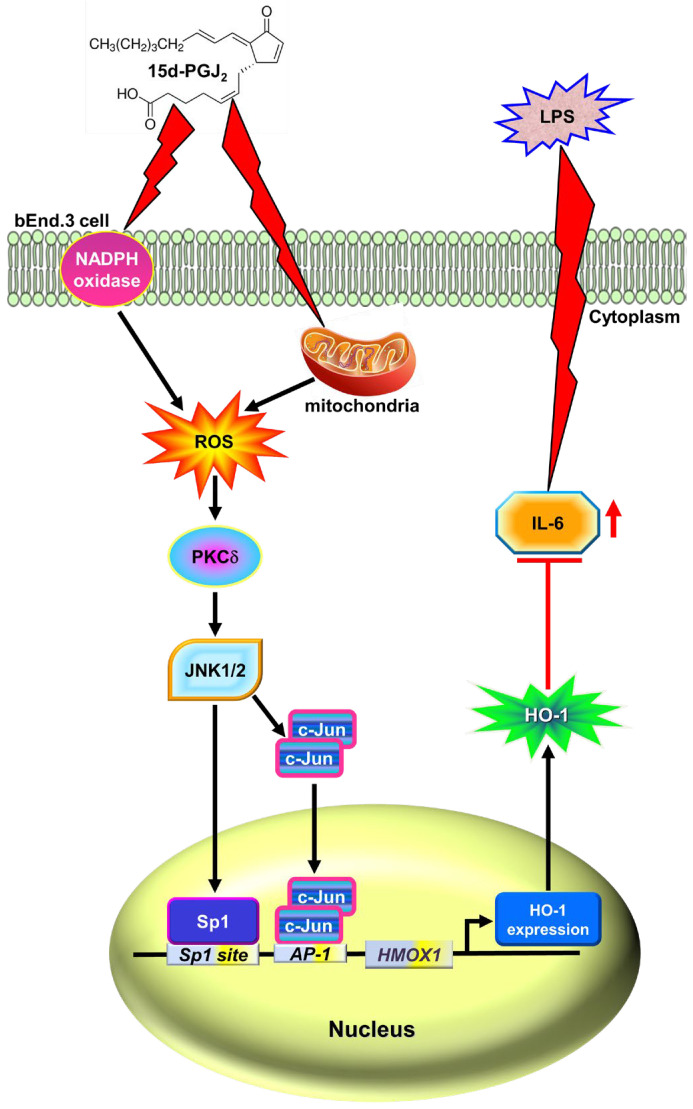
Schematic signaling pathways are involved in 15d-PGJ_2_-induced HO-1 expression protecting against LPS-induced IL-6 secretion in bEnd.3 cells. 15d-PGJ_2_ stimulates NOX- and mitochondria-derived ROS generation, which sequentially activates PKCδ, JNK1/2, and transcription factor Sp1 or c-Jun activity. Activated Sp1 and c-Jun are translocated into the nucleus and then bind with respective Sp1 and AP-1 binding sites on the HO-1 promoter, leading to the induction of HO-1 expression. Moreover, upregulation of HO-1 by 15d-PGJ_2_ attenuates LPS-induced IL-6 expression and secretion, which could protect against inflammatory responses in bEnd.3 cells. Abbreviations: *HMOX1*: heme oxygenase 1 gene; Mito: mitochondria. ┴: inhibition; ↑: activation/increase.

A growing body of evidence indicates that several molecules, such as 15d-PGJ_2_, can produce HO-1, which is important for antioxidant and anti-inflammatory effects [40,41]. Previous studies have indicated that Nrf2 is involved in 15d-PGJ_2_-induced HO-1 expression [27,28]. In the present study, 15d-PGJ_2_-stimulated HO-1 expression is expanded and, at least in part, mediated through the activation of either Sp1 or AP-1 transcription activity dependent on a ROS/PKCδ/JNK1/2 cascade in mouse brain endothelial cells. This report also clarifies that 15d-PGJ_2_ protects brain endothelial cells against LPS-induced IL-6 release via the upregulation of the anti-inflammatory molecule HO-1. Moreover, the mechanisms of HO-1 upregulation by 15d-PGJ_2_ are, at least in part, mediated through mitochondria- or NOX-derived ROS generation-dependent activation of the PKCδ/JNK1/2 cascade, leading to activating Sp1 and c-Jun in bEnd.3 cells (Figure 8).

The results of this study demonstrated that the levels of protein and mRNA expression of HO-1 were significantly induced by 15d-PGJ_2_ in bEnd.3 cells. ROS are created by various chemical processes and enzymatic reactions. Most endogenous sources of ROS come from NOX, mitochondrial electron transport chain, endoplasmic reticulum stress, and oxidative enzymes. The signaling of NOX/ROS is associated with the regulation of cell-related pathways to the expression of various pro-inflammatory mediators, such as IL-6, IL-1, and TNF-α [42]. In contrast, ROS are also well-known as second messengers to protect cells against oxidative stress by maintaining the homeostasis of cellular redox. Our previous studies have demonstrated that NOX/ROS takes part in the induction of HO-1 by various inducers in various types of cells [43,44,45]. In this study, our data also consistently showed that NOX-derived ROS participated in 15d-PGJ_2_-stimulated HO-1 upregulation in bEnd.3 cells, which were mitigated by pretreating cells with either DPI or MitoTempo. These results elucidate that the NOX and mitochondrial sources of ROS are involved in 15d-PGJ_2_-induced HO-1 induction in bEnd.3 cells.

ROS could act as second messengers that further activate various protein kinases, including PKCs. The PKCs belong to serine/threonine kinases, which control a variety of cellular functions, including proliferation, cell survival, and death. A growing body of evidence shows that PKCδ, in particular, contributes to the cellular homeostatic responses against hypoxic stress [46]. The generation of ROS has been uncovered to be able to activate PKCδ, in turn leading to the expression of HO-1. For example, Lee and Yang found that PKCδ translocation triggered by 2,3,7,8,-tetrachlorodibenzo-p-dioxin was mediated through a ROS-dependent pathway in rabbit articular chondrocytes [47]. Fisetin mediated the upregulation of HO-1 in human umbilical vein endothelial cells to protect against oxidative stress, which was blocked by pharmacological inhibitors and PKCδ siRNA [48]. In another study, acrolein enhanced the induction of HO-1, which was also mitigated by Rottlerin, a selective PKCδ inhibitor, and PKCδ siRNA in human bronchial epithelial cells [49]. Consistent with these reports, our findings found that 15d-PGJ_2_-stimulated HO-1 upregulation was also mediated through activating PKC-δ, which was blocked by a selective PKCδ inhibitor, Rottlerin. This finding suggests that PKCδ takes part in 15d-PGJ_2_-induced HO-1 expression, which is further supported by the transfection with PKCδ siRNA. We also noted that 15d-PGJ_2_ is one of the natural PPAR-γ agonists. Our recent report also uncovered that PPAR-γ agonist rosiglitazone enhanced HO-1 expression via PKCα activation in alveolar epithelial cells of the human lung [50]. This difference may be due to the various isoforms of PKCs expressed on these different types of cells and/or different experimental conditions.

JNK1/2 is one member of the MAPK family, which regulates various cellular functions, including the expression of various proteins. A previous study from our team revealed that JNK1/2 has a vital function in CO-releasing molecule (CORM)-2 related effects on human cardiomyocytes, which protects against cardiomyocyte hypertrophy [51]. In addition, our recent report found that JNK1/2-dependent activation of c-Jun participates in the upregulation of HO-1 triggered by Mevastatin, leading to suppression of TNFα-mediated vascular cell adhesion protein 1 expression in the alveolar epithelial cells of the human lung [45]. However, Lim et al. proved that 15d-PGJ_2_ stimulated HO-1 upregulation via p38 MAPK activation and the ROS pathway but not JNK1/2 to activate Nrf2 in vascular smooth muscle cells [28]. Alvarez-Maqueda et al. also indicated that ROS generated through xanthine oxidase and Fenton reactions, p38 MAPK activation, and nuclear factor-κB but not PPAR-γ is involved in 15d-PGJ_2_-induced HO-1 expression in human lymphocytes [52]. Koyani et al. also indicated that 15d-PGJ_2_ induces the expression of HO-1 mediated through the ROS synthesis-dependent p38 MAPK/Akt/Nrf2-Egr1 cascade in the MG-63 cell line [27]. Some of the discrepancies between these findings and our current results may be due to differences in the types of cells and/or experimental conditions. Additionally, several pieces of evidence have indicated that 15d-PGJ_2_ could activate JNK1/2 activity and ROS generation to exert multiple effects on various types of cells [38,53]. Our data consistently demonstrated that JNK1/2 plays an important role in the upregulation of HO-1 by 15d-PGJ_2_, which was regulated by the phosphorylation of JNK1/2. The function of JNK1/2 on HO-1 induction was supported by the inhibition of SP600125 or transfecting cells with JNK2 siRNA, which attenuated the HO-1 upregulation in these cells. These findings suggested that JNK1/2 is involved in 15d-PGJ_2_-stimulated HO-1 induction in bEnd.3 cells.

Previous studies have indicated that transcription factors Sp1 and AP-1 have a vital function in regulating HO-1 expression in in vitro and in vivo studies [54,55,56]. Thus, our study focused on transcription factors, including Sp1 and c-Jun (a subunit of AP-1), to dissect their roles in the regulation of gene expression of HO-1 in bEnd.3 cells. In this study, we found that 15d-PGJ_2_-stimulated HO-1 upregulation is mediated through Sp1 and c-Jun activation. Moreover, the PKCδ/JNK1/2 signaling pathway can regulate 15d-PGJ_2_-induced phosphorylation of Sp1 and c-Jun, contributing to the upregulation of HO-1 expression. A previous study observed that the PKC-ζ pathway resulted in increased activity of Sp1 and upregulation of HO-1 [57]. Our results revealed that in bEnd.3 cells, activation of PKCδ leads to the activation of Sp1 activity and the upregulation of HO-1. Our previous report also indicated that HO-1 upregulation exerts a cytoprotective effect mediated through a p38 MAKP and JNK1/2/Sp1-dependent cascade in human cardiomyocytes stimulated with CORM-2 [51]. The vascular endothelial dysfunction can be improved by JNK/c-Jun-pathway-mediated HO-1 expression with the intervention of Tongxinluo [58]. In addition, we also uncovered that Erk1/2-dependent signaling activates c-Jun and Sp1 to cooperate with Nrf2, leading to increased expression of HO-1 in rat brain astrocytes stimulated with CORM-2 [59]. The current study also uncovered that JNK1/2 regulates HO-1 induction mediated through the transcription factors Sp1 and c-Jun activation. Phosphorylation of Sp1 was blocked by pretreatment with SP600125 and Mithramycin A; the latter had no effect on the phosphorylation of JNK1/2. In addition, the phosphorylation of c-Jun was attenuated by pretreatment with SP600125 or transfection of JNK2 siRNA. These findings indicate that JNK1/2 is an upstream component of Sp1 and c-Jun. Moreover, Sp1 and c-Jun binding with the promoter of HO-1 were also inhibited by pretreatment with SP600125 (Figure 5 and Figure 6). Some of the discrepancies between these findings and our current results may be due to differences in stimuli, cells, and/or experimental conditions. Taken together, Sp1 and c-Jun were, at least partially, activated by the signal transduction of PKCδ/JNK1/2 by 15d-PGJ_2_, leading to the induction of HO-1. Based on the present findings, we clarify that 15d-PGJ_2_ induces HO-1 gene expression via the Sp1 and AP-1 (c-Jun) activation in mouse microvascular bEnd.3 cells.

Endothelial cells are essential for cell–cell adhesion and the formation of the BBB. Therefore, there is considerable interest in the specific characteristics of endothelial cells during the progression of brain pathologies. To study the anti-inflammatory effects of the HO-1 expression induced by15d-PGJ_2_, LPS was used to mimic the cellular inflammation. Some previous reports have indicated that stimulation of LPS can induce IL-6 release. Cytokines have been found to play a broader role in pathology, which are initially thought to be components of the immune system. The levels of cytokines, including TNF, IL-6, and IL-1β, have been demonstrated to be increased in the CNS after injury or inflammation [60]. The previous study showed that IL-6 may affect neuronal function in Parkinson’s disease patients who have higher IL-6 expression in ventricular cerebrospinal fluid [61]. Thus, IL-6 has a crucial function in the development of inflammatory responses that may increase BBB permeability and destroy tight junctions [60]. 15d-PGJ_2_ has been proved to alleviate acute liver injury induced by ConA in mice via upregulating HO-1 and to reduce autophagy in hepatic cells [33]. Moreover, other studies have demonstrated that 15d-PGJ_2_ is beneficial for anti-inflammation, such as inhibition of the LPS-induced TNFα expression [62]. Consistent with these reports, in our model, we revealed that the IL-6 mRNA expression and secretion induced by LPS were, at least partially, inhibited by 15d-PGJ_2_-induced HO-1 upregulation. Therefore, 15d-PGJ_2_-induced HO-1 can be considered an effective intervention for anti-inflammation effects on brain diseases.

## 5. Conclusions

In summary, our results propose that 15d-PGJ_2_-induced expression of HO-1 is regulated by the activation of the NOX- and mitochondria-derived ROS/PKCδ/JNK1/2 signaling pathway to activate Sp1 and c-Jun activities in bEnd.3 cells. In addition, HO-1 upregulation in bEnd.3 cells finally contribute to the 15d-PGJ_2_-promoted anti-inflammatory effects, which can inhibit the LPS-triggered expression and secretion of IL-6 (Figure 8). These results provide new insights into the mechanisms of 15d-PGJ_2_-induced HO-1 and support the hypothesis that 15d-PGJ_2_ may contribute to protecting against LPS-mediated brain inflammation. The lack of animal models to verify in vitro results is the limitation of this study. In order to better interpret the effects of 15d-PGJ_2_, it is worth performing additional in vivo experiments using animal models of neuroinflammation. This study improves the understanding of the mechanisms underlying HO-1 inducers when exerting their potential in the induction of antioxidant enzymes, including HO-1, and the effects of anti-inflammation, which may be beneficial for treating neurodegenerative diseases. Moreover, a better understanding of how 15d-PGJ_2_ regulates LPS-induced pro-inflammatory mediators may also create opportunities for the development of potential anti-inflammatory therapeutics for neuroinflammation and brain disorders.

## Data Availability

All of the data is contained within the article.

## Abbreviations

AP-1: activator protein 1; BBB: blood–brain barrier; bEnd.3: mouse brain microvascular endothelial; CNS: central nervous system; CO: carbon monoxide; COX: cyclooxygenase; DCF: 2′-7′dichlorofluorescein; DMEM: Dulbecco’s modified Eagle’s medium; 15d-PGJ_2_: 15-deoxy-Δ^12,14^-prostaglandin J_2_; DPI: diphenyleneiodonium chloride; FBS: fetal bovine serum; GAPDH: glyceraldehyde-3-phosphate dehydrogenase; H_2_DCFDA: 2′,7′-dichlorodihydrofluorescein diacetate; HO-1: heme oxygenase-1; JNK: c-Jun N-terminal kinases; MAPK: mitogen-activated protein kinase; MOI: multiplicity of infection; NOX: NADPH oxidase; PBS: phosphate-buffered saline; PG: prostaglandins; PKC: protein kinase C; PPAR: peroxisome proliferator-activated receptor; ROS: reactive oxygen species; Sp1: specificity protein 1; TNF: tumor necrosis factor; TTBS: Tween Tris-buffered saline.
